# Matrix Metalloproteinase Triple-Helical Peptide Inhibitors: Potential Cross-Reactivity with Caspase-11

**DOI:** 10.3390/molecules24234355

**Published:** 2019-11-28

**Authors:** Anna M. Knapinska, Melissa Hart, Gary Drotleff, Gregg B. Fields

**Affiliations:** 1 Department of Chemistry & Biochemistry, Florida Atlantic University, 5353 Parkside Drive, Jupiter, FL 33458, USA; ania.knapinska@fau.edu (A.M.K.); melissahart2015@fau.edu (M.H.); gdrotlef@fau.edu (G.D.); 2 Department of Chemistry, The Scripps Research Institute/Scripps Florida, 130 Scripps Way, Jupiter, FL 33458, USA

**Keywords:** matrix metalloproteinase, caspase, sepsis, phosphinate, protease inhibitor, triple-helical peptide inhibitor

## Abstract

Triple-helical peptide inhibitors (THPIs) of matrix metalloproteinases (MMPs) have recently been demonstrated to be effective in a variety of animal models of disease, coincidental with knockout studies. However, passenger mutations have been described in MMP knockout mice that impact the activity of other proteins, including caspase-11. Thus, it is possible that the results observed with THPIs may be based on inhibition of caspase-11, not MMPs. The present study evaluated whether THPIs were cross-reactive with caspase-11. Two different THPIs were tested, one that is known to inhibit MMP-1 and MMP-8 (GlyΨ{PO_2_H-CH_2_}Ile-His-Lys-Gln THPI) and one that is selective for MMP-2 and MMP-9 (α1(V)GlyΨ{PO_2_H-CH_2_}Val [mep_14,32_,Flp_15,33_] THPI). No inhibition of caspase-11 was observed with GlyΨ{PO_2_H–CH_2_}Ile–His–Lys–Gln THPI, even at an inhibitor concentration of 5 μM, while 5 μM α1(V)GlyΨ{PO_2_H-CH_2_}Val [mep_14,32_,Flp_15,33_] THPI exhibited 40% inhibition of caspase-11. Further testing of GlyΨ{PO_2_H-CH_2_}Ile-His-Lys-Gln THPI revealed nM inhibition of MMP-2, MMP-9, and MMP-13. Thus, the effectiveness of GlyΨ{PO_2_H-CH_2_}Ile-His-Lys-Gln THPI observed in a sepsis animal model may not be due to caspase-11 inhibition, but may be due to broader MMP inhibition than previously thought.

## 1. Introduction

The matrix metalloproteinase (MMP) family of enzymes has been implicated in a great variety of diseases [1,2,3,4,5,6], resulting in the pursuit of MMP inhibitors that has spanned decades [1,7,8,9,10,11,12]. A variety of phosphorus-based peptides have been developed as MMP inhibitors [7,13,14]. Phosphinates/phosphinic peptides contain a hydrolytically stable, tetrahedral phosphinic pseudo-dipeptide moiety which mimics the tetrahedral intermediate formed during Zn^2+^ metalloproteinase-catalyzed amide bond hydrolysis. We have reported that phosphinic triple-helical peptides (THPs) behave as effective transition state analog inhibitors of collagenolytic MMPs [15,16,17,18,19]. Recently, the triple-helical peptide inhibitor (THPI) (Gly-Pro-Hyp)_4_-Gly-mep-Flp-Gly-Pro-Gln-{GlyΨ(PO_2_H-CH_2_)Ile}-His-Lys-Gln-Arg-Gly-Val-Arg-Gly-mep-Flp-(Gly-Pro-Hyp)_4_-Tyr-NH_2_ (designated GlyΨ{PO_2_H-CH_2_}Ile-His-Lys-Gln THPI) (where mep = (*2S,4R*)-4-methylproline, Flp = (*2S,4R*)-4-fluoroproline, and Hyp = 4-hydroxy-l-proline) was shown to inhibit MMP-8 in the nanomolar range while being a much weaker inhibitor of MT1-MMP [19]. Gene ablation studies have indicated that MMP-8 facilitates sepsis in animal models, while MT1-MMP is protective [19,20,21]. GlyΨ{PO_2_H-CH_2_}Ile-His-Lys-Gln THPI was thus applied in an animal model of sepsis [19]. Using the cecal ligation and puncture (CLP) sepsis model, 70% of the wild-type mice treated with GlyΨ{PO_2_H-CH_2_}Ile-His-Lys-Gln THPI survived after 7 days, while none of the non-treated wild-type mice did [19].

Genetically modified (knockout) mice have been utilized to delineate the roles of MMPs in normal physiological function and pathological states. To examine the specificity of the GlyΨ{PO_2_H-CH_2_}Ile-His-Lys-Gln THPI, *Mmp8* null mice underwent CLP and were then either treated with the THPI or not treated. A survival advantage during sepsis was observed for *Mmp8* null mice compared with wild-type mice [19]. Survival of THPI-treated wild-type mice mirrored that of non-treated *Mmp8* null mice, while survival of *Mmp8* null mice was not augmented by inhibitor treatment [19]. Thus, in consideration of the *Mmp8* null mice data, the GlyΨ{PO_2_H-CH_2_}Ile-His-Lys-Gln THPI was deemed as acting specifically towards MMP-8 in vivo.

The identification of passenger mutations that can accompany MMP knockouts has raised serious concerns as to the interpretation of results from disease models in which MMPs were implicated [22]. For example, *Mmp7*, *Mmp8*, or *Mmp13* null mice were found to be protected from lipopolysaccharide (LPS) lethality (septic shock) [23,24,25]. However, these knockout mice carried a passenger mutation that inactivated *Casp11* (the mouse ortholog of human *Casp4* and *Casp5*) [22]. Caspase-11 has been demonstrated to participate in LPS-induced endotoxic shock and subsequent lethality [26,27]. Mice possessing a non-functional caspase-11 are more resistant to LPS-induced septic shock [28], and the passenger mutation of *Casp11* resulted in mice resistant to LPS-induced endotoxic shock [22,29].

In the above example, the results from MMP-8 knockout mice appear to be validated through the use of a THPI which targeted the MMP of interest (MMP-8) in wild-type mice, as the same phenotype was observed for the CLP knockout mice and the CLP wild type mice treated with the GlyΨ{PO_2_H-CH_2_}Ile-His-Lys-Gln THPI. However, if the applied THPI non-specifically inhibited other enzymes, interpretation of the results becomes ambiguous. Given that the *Mmp8* null mice may have had a *Casp11* inactivating mutation [22], the mirroring of survival in the *Mmp8* null mice by the inhibitor treated wild-type mice could have been the result of the THPI inhibiting caspase-11 in the wild-type mice. MMP inhibitors are not anticipated to inhibit caspase-11, due to the different active site chemistries and sequence specificities [30,31,32,33,34]. However, recent research has indicated that caspase-11 recognition of substrates can be strongly influenced by motifs outside of the active site [34], and thus, there is a possibility of non-specific inhibition by MMP inhibitors whose structures may be complimentary to caspase-11 motifs. In addition, MMP inhibitors that are designed to interact with the active site Zn^2+^ can inhibit non-MMP activity by non-selective metal binding [35,36]. The present study has examined the inhibition of (a) caspase-11 by two phosphinate-based THPIs and (b) other collagenolytic MMPs by GlyΨ{PO_2_H-CH_2_}Ile-His-Lys-Gln THPI.

## 2. Results

**Figure 1:**
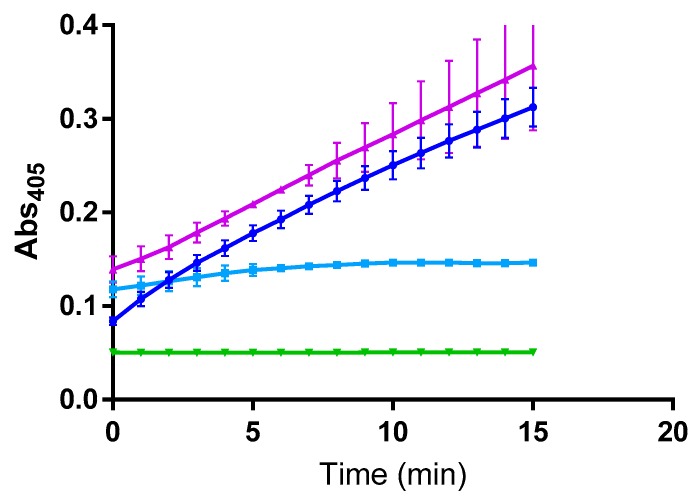
Effect of inhibitors on caspase-11 activity. Hydrolysis of acetyl-Trp-Glu-His-Asp-pNA by caspase-11 (dark blue) and inhibition by 5 μM acetyl-Leu-Glu-Val-Asp-CHO (light blue) or 5 μM GlyΨ{PO_2_H-CH_2_}Ile-His-Lys-Gln THPI (purple). Acetyl-Trp-Glu-His-Asp-pNA alone (green) was used as a control.

Caspase-11 hydrolysis of acetyl-Trp-Glu-His-Asp-pNA was examined at several enzyme and substrate concentrations to obtain conditions under which enzyme inhibition could be studied. It was ultimately determined that 3 U/μL (1.08 μM) caspase-11 and 250 μM acetyl-Trp-Glu-His-Asp-pNA provided a reasonably linear rate of hydrolysis over 15 min. Acetyl-Leu-Glu-Val-Asp-CHO was incubated with caspase-11 at a concentration of 5 μM for 2 h prior to the addition of substrate, and was found to completely inhibit enzymatic activity (Figure 1).

The potential inhibition of caspase-11 by GlyΨ{PO_2_H-CH_2_}Ile-His-Lys-Gln THPI was examined by adding 5 μM of the inhibitor to the enzyme for 2 h prior to addition of substrate. A 2 h incubation was utilized based on (a) the generally observed behavior of slow on and off rates for tight-binding inhibitors [37], (b) studies demonstrating that high affinity phosphinate inhibitors of Zn^2+^ metalloproteinases are slow binding [38], and (c) our prior studies using THPIs [39]. This concentration was comparable to that used in our prior CLP mouse model studies (13.5 μM) [19]. No inhibition of caspase-11 activity was observed over 15 min (Figure 1).

**Figure 2:**
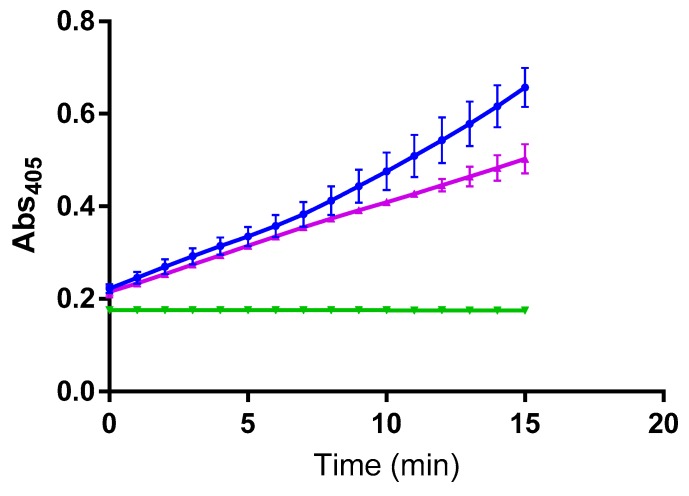
Effect of α1(V)GlyΨ{PO_2_H-CH_2_}Val [mep_14,32_,Flp_15,33_] THPI on caspase-11 activity. Hydrolysis of acetyl-Trp-Glu-His-Asp-pNA by caspase-11 (dark blue) and inhibition by 5 μM α1(V)GlyΨ{PO_2_H-CH_2_}Val [mep_14,32_,Flp_15,33_] THPI (purple). Acetyl-Trp-Glu-His-Asp-pNA alone (green) was used as a control.

A second THPI was examined for inhibitory potential towards caspase-11. (Gly-Pro-Hyp)_4_-Gly-mep-Flp-Gly-Pro-Pro-GlyΨ{PO_2_H-CH_2_}Val-Val-Gly-Glu-Gln-Gly-Glu-Gln-Gly-Pro-Pro-Gly-mep-Flp-(Gly-Pro-Hyp)_4_-NH_2_ (designated α1(V)GlyΨ{PO_2_H-CH_2_}Val [mep_14,32_,Flp_15,33_] THPI) is an effective, and selective, inhibitor of MMP-2 and MMP-9, with K_i_ values of 2.24 and 0.98 nM at 37 °C for MMP-2 and MMP-9, respectively, and exhibiting no or weak (K_i_ = 10–50 μM) inhibition of MMP-1, MMP-3, MMP-8, MMP-13, and MT1-MMP [15,19]. α1(V)GlyΨ{PO_2_H-CH_2_}Val [mep_14,32_,Flp_15,33_] THPI was recently shown to be efficacious in an animal model of multiple sclerosis [19]. 5 μM of α1(V)GlyΨ{PO_2_H-CH_2_}Val [mep_14,32_,Flp_15,33_] THPI was added to the enzyme for 2 h prior to addition of substrate. Approximately 40% inhibition of caspase-11 activity was observed after 15 min (Figure 2). The extent of this inhibition did not increase after 1 h (data not shown). We examined the inhibition of caspase-11 by the α1(V)GlyΨ{PO_2_H-CH_2_}Val [mep_14,32_,Flp_15,33_] THPI using no inhibitor pre-incubation time, and found no inhibition to occur at early time points (data not shown), in similar fashion to what was observed for a 2 h inhibitor pre-incubation (Figure 2).

The inhibitory potency of GlyΨ{PO_2_H-CH_2_}Ile-His-Lys-Gln THPI was initially examined for MMP-1, MMP-3, MMP-8, and MT1-MMP (Table 1) [19]. Selectivity within the collagenolytic MMPs was further examined by treating MMP-2, MMP-9, and MMP-13 with GlyΨ{PO_2_H-CH_2_}Ile-His-Lys-Gln THPI. All three enzymes were inhibited by the THPI (Table 1). The K_i_ value for MMP-13 was better than that observed for MMP-8 (Table 1). MMP-2 and MMP-9 were very efficiently inhibited by GlyΨ{PO_2_H-CH_2_}Ile-His-Lys-Gln THPI, with K_i_ values below 10 nM (Table 1).

## 3. Discussion

The *Mmp8* null mice utilized in our CLP study were on a C57BL/6J background [19], which may still harbor the *Casp11* mutation depending upon the extent of backcrossing [22]. The inhibition of caspase-11 by a THPI could occur by two non-exclusive mechanisms: (a) chelation of a caspase-11 metal ion by the THPI phosphinate; and (b) binding of the THPI sequence to a complementary motif on the surface of caspase-11. To consider possibility (a), the mechanism by which caspase-11 contributes to CLP/LPS-induced lethality needs to be taken into account. The CLP sepsis model induces polymicrobial peritonitis and results in translocation of bacteria into the bloodstream (bacteremia) [40,41]. Both Gram negative and Gram positive bacteria enter the bloodstream and tissues, with a shift to Gram negative bacteria with disease progression [40]. Gram negative, but not Gram positive, bacteria activate the caspase-11 non-canonical inflammasome pathway [28]. Caspase-11 directly binds the lipid A tail of LPS via the caspase recruitment domain motif [28]. LPS binding first results in the formation of caspase-11 dimers, followed by auto-hydrolysis at the interdomain linker residue Asp285 [42]. Caspase-11 then undergoes oligomerization, ultimately inducing pyroptosis and secretion of the pro-inflammatory cytokines interleukin-1β and interleukin-18 [28].

MMP inhibitors have an unfortunate history of non-selectivity. Of specific concern is the ability of zinc binding groups to behave in a non-selective fashion. For example, hydroxamic acids have been commonly used in MMP inhibitors, but the ability of hydroxamic acids to non-specifically chelate metal ions, in some cases with greater affinity than zinc, has been noted [35,36]. Caspase-11 is a Cys protease, and it is not anticipated that non-selective metal chelation would be a concern as caspase family members caspase-1, caspase-2, caspase-3, caspase-7, caspase-8, and caspase-9 do not possess structural metals [43,44]. However, the human orthologs of caspase-11, caspase-4 and caspase-5, have been reported to each have one Mg^2+^ ion present (https://swissmodel.expasy.org/repository/uniprot/P49662 and https://swissmodel.expasy.org/repository/uniprot/P51878). Homology modeling indicated that in caspase-4 the Mg^2+^ binds to His338 of the A chain, His338 of the B chain, and Tyr370 of the B chain, while in caspase-5, the Mg^2+^ binds to His395 of the A chain and Glu398 of the B chain. Thus, the Mg^2+^ ion could stabilize caspase-11 dimers, and dimerization is an important step in the caspase-11 pathway contributing to endotoxic shock (see above). As indicated by studies with nucleic acids, phosphates will readily bind Mg^2+^ [45,46]. The GlyΨ{PO_2_H-CH_2_}Ile-His-Lys-Gln THPI did not show inhibition of caspase-11, and thus, Mg^2+^ ions most likely do not undergo non-specific chelation by THPIs (see above).

In consideration of mechanism (b), THPIs have extended sequences that interact with regions well outside of the active site in MMPs [16,17]. These regions of interaction are referred to as secondary binding sites (exosites). The THPI extended sequences could interact with caspase-11 exosites. It has been proposed that caspase-11 substrate specificity is governed by exosite interactions, as the substrate preferences of caspase-11 cannot be explained by the sequences at the active site [34]. One possibility is that negatively charged residues in the P_7_-P_10_ region may be unfavorable for substrate interaction with caspase-11 [34]. GlyΨ{PO_2_H-CH_2_}Ile-His-Lys-Gln THPI possesses no negative charges, and thus could interact with caspase-11. However, as this THPI did not inhibit caspase-11, we can assume that there is no exosite interaction.

It is interesting to note that α1(V)GlyΨ{PO_2_H-CH_2_}Val [mep_14,32_,Flp_15,33_] THPI exhibited some inhibition of caspase-11 activity, although it occurred at a relatively high inhibitor concentration (5 μM). It is not likely that this inhibition was due to Mg^2+^ chelation (based on the lack of caspase-11 inhibition by GlyΨ{PO_2_H-CH_2_}Ile-His-Lys-Gln THPI). This suggests a possible interaction between α1(V)GlyΨ{PO_2_H-CH_2_}Val [mep_14,32_,Flp_15,33_] THPI and a caspase-11 allosteric site, although α1(V)GlyΨ{PO_2_H-CH_2_}Val [mep_14,32_,Flp_15,33_] THPI possesses 6 negatively charged residues, which are not favored in the P_7_-P_10_ region by caspase-11 (see above).

There are two caveats to the interpretation of results from the present study. First, the caspase-11 substrate used here is small (4 amino acids), whereas in the CLP/LPS animal models caspase-11 is processing proteins. An exosite-binding inhibitor might not impact caspase-11 hydrolysis of a small substrate but could impact processing of a protein, unless binding to the exosite had an allosteric effect on the enzyme. Second, chelation of Mg^2+^ ions might not impact caspase-11 activity towards a small substrate, but rather inhibit dimerization of the enzyme. Dimerization might be required to facilitate specific proteolytic activities in CLP/LPS models (see above).

We presently observed that GlyΨ{PO_2_H-CH_2_}Ile-His-Lys-Gln THPI did not inhibit caspase-11 activity. This result supports our prior report on the modulation of sepsis by MMP-8 inhibition, not caspase-11 inhibition [19]. However, our prior studies did not examine the full range of collagenolytic MMPs that might have been inhibited by GlyΨ{PO_2_H-CH_2_}Ile-His-Lys-Gln THPI. We found that MMP-2, MMP-9, and MMP-13 are effectively inhibited by the THPI, along with MMP-8. Thus, the observed protection from sepsis in the CLP mouse model by GlyΨ{PO_2_H-CH_2_}Ile-His-Lys-Gln THPI may have been due to inhibition of multiple MMPs. The use of more selective inhibitors will elucidate the role of each MMP in sepsis.

## 4. Materials and Methods

GlyΨ{PO_2_H-CH_2_}Ile-His-Lys-Gln THPI and α1(V)GlyΨ{PO_2_H-CH_2_}Val [mep_14,32_,Flp_15,33_] THPI were synthesized and characterized as described previously [19]. Recombinant caspase-11 (residues 81-373), caspase substrate acetyl-Trp-Glu-His-Asp-pNA (where pNA = *p*-nitroaniline) (MW 747.8 Da), and caspase-4 and caspase-5 inhibitor acetyl-Leu-Glu-Val-Asp-CHO (MW 500.6 Da) were obtained from Enzo Life Sciences, Inc.

### 4.1. Caspase Enzyme Assay

Caspase-11 (9 U/μL in 10 μL caspase activity buffer (100 mM 2-(*N*-morpholino)ethanesulfonic acid (MES)), pH 6.5, 0.1% 3-[(3-cholamidopropyl)dimethylammonio]-1-propanesulfonate (CHAPS), 10% poly(ethylene-glycol) (PEG)-8000, 10 mM dithiothreitol (DTT)) was added to 10 μL of caspase-11 inhibitor (5 μM), THPI (5 μM), or no inhibitor in caspase activity buffer. For testing THPIs as inhibitors, enzyme was incubated with THPIs for 2 h at 37 °C. Residual enzyme activity was monitored by adding 10 μL of caspase substrate in caspase activity buffer to produce a final concentration of <0.1K_M_. pNA cleavage from the peptide was monitored at 37 °C over 1 h using λ = 405 nm on a Bio-Tek Synergy H1 Reader or H4 Hybrid Reader.

### 4.2. Matrix Metalloproteinases (MMPs)

Human recombinant, full-length proMMP-8 and proMMP-13 were purchased from R&D Systems (Minneapolis, MN, USA) and activated according to the manufacturer’s instructions prior to use. *p*-Aminophenylmercuric acetate was purchased from EMD Biosciences (San Diego, CA, USA), and trypsin (TPCK-treated) was purchased from Worthington Biochemical Corporation (Lakewood, NJ, USA). Recombinant MMP-2 and MMP-9 catalytic domain were purchased from Sigma-Aldrich (St. Louis, MO, USA) and used directly without further activation in the desired concentration.

ProMMP-8 and proMMP-13 were activated by mixing an equal volume of stock enzyme solution and activator (*p*-aminomercuric acetate to a final concentration of 1 mm) followed by 2-3 h incubation in a 37 °C water bath. Activated MMPs were diluted to 200-400 nm in ice cold TSB*Zn (50 mm Tris, 100 mm NaCl, 10 mm CaCl_2_, 0.05% Brij-35, 0.02% NaN_3_, 1 μm ZnCl_2_, pH 7.5) to prevent autoproteolysis. Enzyme aliquots were either kept on wet ice and used the same day or immediately frozen at −80 °C. MMP activity was initially evaluated by using the Knight substrate (Mca-Lys-Pro-Leu-Gly-Leu-Lys-(Dnp)-Ala-Arg-NH_2_, where Mca = 7-methoxycoumarin-4-acetyl and Dnp = 2,4-dinitrophenyl) and compared with prior data [16,17]. In this way, activity towards the substrate was used as an indicator of enzyme integrity, rather than TIMP titration, as performed previously [47].

### 4.3. Inhibition Kinetic Studies

Peptide substrate and THPI solutions were prepared using TSB*Zn. 1–10 nM enzyme was incubated with varying concentration of inhibitor for 2 h at 37 °C. Residual enzyme activity was monitored by adding Knight substrate solution in TSB*Zn to produce a final concentration of <0.1K_M_. Initial velocity rates were determined from the first 10 min of hydrolysis when product release is linear with time. Fluorescence was measured on a Bio-Tek Synergy H1 Reader or H4 Hybrid Reader using λ_excitation_ = 324 nm and λ_emission_ = 393 nm. Apparent K_i_ values were calculated from the following formulas [15]:

*v*_i_/*v*_o_ = {*E*_t_ – *I*_t_ – K_i_^(app)^ + ((*E*_t_ – *I*_t_ – K_i_^(app)^)^2^ + 4*E*_t_K_i_^(app)^)^0.5^}/2*E*_t_ (1)

K_i_^(app)^ = K_i_({*A*_t_ + K_M_}/K_M_) (2)

where *I*_t_ is the total inhibitor concentration, *E*_t_ is the total enzyme concentration, *A*_t_ is the total substrate concentration, *v*_o_ is the activity in the absence of inhibitor, and K_M_ is the Michaelis constant. In our assays the value of *E*_t_/K_i_^(app)^ does not exceed 100 so that the inhibitor is distributed in both free and bound forms, and K_i_^(app)^ can be calculated by fitting inhibition data to equation 1. Because the substrate concentration is less than K_M_/10, K_i_^(app)^ values are insignificantly different from true K_i_ values. In cases where weak inhibition occurred, K_i_^(app)^ values were calculated using Prism 7.0 (GraphPad, San Diego, CA, USA) by fitting data to the equation *v*_i_ = *v*_o_/(1 + *I*_t_/ K_i_^(app)^).

## Figures and Tables

**Table 1:** Inhibition of matrix metalloproteinases (MMPs) by GlyΨ{PO_2_H-CH_2_}Ile-His-Lys-Gln THPI at 37 °C.

Enzyme	K_i_ (nM)
MT1-MMP	4704 ± 708.4 ^1^
MMP-8	124.6 ± 6.9 ^1^
MMP-8	378.1 ± 40.6
MMP-1	169.2 ± 28.4 ^1^
MMP-3	NI ^1,2^
MMP-2	2.2 ± 0.20
MMP-9	3.7 ± 0.40
MMP-13	31.7 ± 7.6

^1^ Previously reported [19]. ^2^ NI = No inhibition at a triple-helical peptide inhibitor (THPI) concentration of 1000 nM.
